# Clinical validation of EDIT-B test for the diagnosis of bipolar disorder

**DOI:** 10.1192/j.eurpsy.2022.1047

**Published:** 2022-09-01

**Authors:** J.-D. Abraham, N. Salvetat, P. Guerra, M. Ferrari, P. Le Guen, O. Biglia, C. Henry, L. Kessing, J.M. Haro, E. Vieta, D. Weissmann

**Affiliations:** 1 Alcediag/Sys2Diag, Neurology, Montpellier, France; 2 Product Life Group, Plg, Suresnes, France; 3 Synlab, Italy, Monza, Italy; 4 Aixial, France, Boulogne-Billancourt, France; 5 Veracyte, Luminy Biotech Entreprises, Marseille, France; 6 Institut Pasteur, Unité Perception Et Mémoire, Paris, France; 7 The Copenhagen Affective Disorder Research Center, Rigshospitalet, Copenhagen, Denmark; 8 Parc Sanitari Sant Joan de Déu, Fundació Sant Joan De Déu, Cibersam, Barcelona, Spain; 9 University of Barcelona, Hospital Clinic, Barcelona, Spain

**Keywords:** bipolar disorder, diagnostic, clinical study, epigenetics

## Abstract

**Introduction:**

Bipolar disorder (BD) is a psychiatric disorder characterized by alternating episodes of high mood and low mood similar to depression. To differentiate BD patients from unipolar (UN) depressed patients remains a challenge and the clinical scales available failed to distinguish these 2 populations. ALCEDIAG developed EDIT-B, the first blood test able to make a differential diagnosis of BD. Based on RNA editing modifications measurement and AI, the test requires a simple blood draw and equipment available in most central laboratories. A first study on 160 UN and 95 BD patients allowed a differential diagnosis with an AUC of 0.935 and high specificity (Sp=84.6%) and sensitivity (Se=90.9%). A multicentric clinical study has been set up to validate these performances.

**Objectives:**

The objective of this project is to run a multicentric clinical study in Europe and assess the performances of the test.

**Methods:**

The EDIT-B project, led by Alcediag, is supported by EIT-Health grant (European institute of Innovation and Technology) and gathers 4 clinical centers in 3 countries (France, Spain, Danemark), a CRO for the clinical study management (Aixial), a CRO for the development of a diagnostic kit (Veracyte), a diagnostic lab for molecular biology analyses (Synlab), and a regulatory company (PLG).

**Results:**

At the end of the study, the EDIT-B performance will be confirmed and the test will be CE-marked.

**Conclusions:**

This test will address the needs of millions of patients suffering from misdiagnosis and therefore allow them to receive the correct treatment.

**Disclosure:**

JDA, NS and DW are employees of Alcediag.

